# Response to: influence of EMS-physician presence on survival after out-of-hospital cardiopulmonary resuscitation

**DOI:** 10.1186/s13054-016-1495-y

**Published:** 2016-10-12

**Authors:** Johannes von Vopelius-Feldt, Jonathan Benger

**Affiliations:** 1 Faculty of Health and Life Sciences, University of the West of England, Glenside Campus, Blackberry Hill, Bristol, BS16 1DD UK; 2 Academic Department of Emergency Care, University Hospitals Bristol NHS Foundation Trust, Upper Maudlin Way, Bristol, BS2 8HW UK

Böttiger et al. [1] present a meta-analysis demonstrating improved outcomes after out-of-hospital cardiac arrest (OHCA) attended by emergency medical services (EMS) physicians, when compared with attendance by paramedics. Because the meta-analysis is based solely on observational studies, we wonder whether a narrative review of the literature would have allowed the reader to reach a more balanced understanding of the available evidence.

There is significant heterogeneity in study sizes, ranging from *n* = 49 to *n* = 95,072. Given that the total number of analysed cases is *n* = 126,829, the study by Yasunaga et al. [2] will inevitably dominate the results.

This is of particular importance for two reasons. Firstly, Yasunaga et al. examined only a subgroup of bystander-witnessed OHCA in Japan. Secondly, EMS physicians in Japan were provided by individual hospitals. The authors point out that ‘hospitals with [EMS]-physicians typically provide more optimal post-return of spontaneous circulation treatments, including therapeutic hypothermia and percutaneous coronary intervention’ [2]. While the study showed significant survival benefit associated with EMS-physician presence, it is unclear whether this benefit occurs due to advanced pre-hospital or in-hospital treatment.

The same limitations apply to the second largest study (*n* = 18,462). Hagihara et al. [3] also utilised the national Japanese OHCA database and found improvements in survival with EMS-physician presence. The authors state that their findings ‘need confirming with consideration of in-hospital treatment’.

These two Japanese studies make up nearly 90 % of the cases included in the meta-analysis. Despite this imbalance, Böttiger et al. did not perform sensitivity analysis excluding these two studies because the remaining studies ‘were largely consistent in effect size’ [1]. However, the effect sizes presented for a number of these studies require careful consideration.

The third-largest study by Fischer et al. [4] (*n* = 4298) is a retrospective analysis of two previous publications, independently describing survival after OHCA in the UK (paramedic-based EMS) and in Germany (physician-based EMS). While survival in Germany was significantly higher, ambulance response times were also shorter in Germany. No information is available on important prognostic factors such as age of patients or percentage of cases with shockable rhythm.

The work by Kojima et al. [5] (*n* = 4144) is a conference abstract presenting limited information. The authors again used the national Japanese OHCA database and the period of data collection overlaps with both Japanese studies described earlier.

We agree with Böttiger et al. that the individual studies included in the meta-analysis represent the best available evidence. However, we suggest that the benefit of EMS physicians attending OHCA remains uncertain.

## Abbreviations

CI: Confidence interval
CPR: Cardiopulmonary resuscitation
EMS: Emergency medical services
OHCA: Out-of-hospital cardiac arrest
OR: Odds ratio
