# Supplementary material for: Response to: influence of EMS-physician presence on survival after out-of-hospital cardiopulmonary resuscitation
Source: Crit Care. 2016 Oct 12;20:324. doi: 10.1186/s13054-016-1495-y (PMC5062867; doi:10.1186/s13054-016-1495-y)
Supplement: Related texts

# Reply: Authors’ response

**Authors:** Bernd W. Böttiger, Michael Bernhard, Jürgen Knapp, Peter Nagele

We thank Dr von Vopelius-Feldt and Dr Benger for their interest and comments on our systematic review and meta-analysis about the positive impact of EMS-physician presence on survival after out-of-hospital cardiopulmonary resuscitation [1].

They correctly mention significant heterogeneity among the study sizes (ranging from *n* = 49 to *n* = 95,072) of patients suffering from OHCA. The pooled sample size with *n* = 126,829 was dominated by two Japanese studies [2, 3], making up nearly 90 % of all cases included in the meta-analysis. They pointed out that we did not perform a sensitivity analysis which excluded these two studies, both from a large, nationwide Japanese database. We did not present a sensitivity analysis due to the fact that all studies were largely consistent in effect size. The pooled OR for survival-to-hospital discharge for all studies was 2.03 (95 % CI: 1.48–2.79). After excluding both Japanese studies from the meta-analysis, the pooled OR for survival-to-hospital discharge was 2.29 (95 % CI: 1.36–3.87) (Fig. 1). The results were therefore consistent whether or not the Japanese studies were included in our meta-analysis.

**Fig. 1:** Survival to hospital discharge comparing EMS-physician CPR with paramedic-guided CPR after excluding the two Japanese studies. *CI* confidence interval, *EMS* emergency medical services

Second, von Vopelius-Feldt and Benger point out that the study by Fischer et al. [4] did not include prognostic factors, such as age of patients or incidence of shockable rhythm. This observation is correct and it is theoretically possible that prognostic factors between both patient populations might differ. Fischer et al.’s study is therefore one of the methodically less valuable studies included in our analysis, but this is one of the often discussed limitations of a meta-analysis.

Third, it is correct that the cited publication by Kojima et al. [5] is a conference abstract. To our knowledge the results were not published in a peer-reviewed journal, but guidelines for systematic reviews strongly recommend the inclusion of all available evidence to reduce publication bias.

A major limitation in this whole scientific field – and as discussed in our publication [1] – is that randomised controlled trails comparing EMS-physician-guided and paramedic-guided CPR in patients suffering from OHCA will not be possible for many reasons. Therefore, all of the available evidence came from observational studies or CPR registries, resulting in an adequate level of evidence. Despite these unavoidable limitations, our systematic review provides the highest and only available evidence for the impressive effectiveness of physician-guided CPR in patients suffering from OHCA today.
